# Ginsenoside Rc Promotes Anti-Adipogenic Activity on 3T3-L1 Adipocytes by Down-Regulating C/EBPα and PPARγ

**DOI:** 10.3390/molecules20011293

**Published:** 2015-01-14

**Authors:** Ji-Won Yang, Sung Soo Kim

**Affiliations:** 1 Department of Food and Nutrition, Kyunghee University, Seoul 130-701, Korea; E-Mail: caroline.yangjiwon@gmail.com; 2 Division of Strategic Food Research, Ginseng Research Center, Korea Food Research Institute, Seongnam 463-746, Korea

**Keywords:** ginsenoside Rc, 3T3-L1, obesity

## Abstract

*Panax ginseng* and its major components, the ginsenosides, are widely used in oriental medicine for the prevention of various disorders. In the present study, the inhibitory activity of ginsenoside Rc on adipogenesis was investigated using the 3T3-L1 cell line. The results obtained showed that Rc reduced the proliferation and viability of 3T3-L1 preadipocytes in a dose-dependent manner. Treatment with Rc decreased the number of adipocytes and reduced lipid accumulation in maturing 3T3-L1 preadipocytes, demonstrating an inhibitory effect on lipogenesis. Moreover, it was found that Rc directly induced lipolysis in adipocytes and down-regulated the expression of major transcription factors of the adipogenesis pathway, such as PPARγ and C/EBPα. These findings indicate that Rc is capable of suppressing adipogenesis and therefore they seem to be natural bioactive factors effective in adipose tissue mass modulation.

## 1. Introduction

*Panax ginseng* C.A. Meyer has been used as an important medicinal herb in traditional Asian medicine since antiquity. The pharmacological activities and chemical components of *P. ginseng* have been widely investigated [1,2]. Ginsenosides, the main active components of *P. ginseng*, have shown various pharmacological actions, such as anti-oxidation, anti-aging, and anti-tumor effects, and modulation of the immune system [3]. More than 40 ginsenosides have been characterized, which can be divided into three categories according to the position of the protopanaxadiol (PPD), protopanaxatriol (PPT), and oleanolic acid side chains on the shared aglycone skeleton [4]. For many years, research on ginsenosides has focused on their structures and biological activities [5,6,7,8,9,10,11]. Ginsenoside Rc, one of the main PPD ginsenosides, has been widely studied for its biological activities [12,13,14,15].

Obesity is a serious health problem because it is implicated in various diseases including type II diabetes, hypertension, coronary heart disease, and cancer [16]. Obesity is characterized by increased adipose tissue mass that results from both increased fat cell number and increased fat cell size [17]. The amount of adipose tissue mass can be regulated by the inhibition of adipogenesis from fibroblastic preadipocytes to mature adipocytes [18] and induction of apoptosis [19] in adipose tissues. Mature adipocytes differentiate from fibroblast-like preadipocytes, mainly under the control of two key families of adipogenic transcription factors, CCAAT/enhancer binding protein (C/EBP) and Peroxisome proliferator-activated receptor-γ (PPARγ) [20]. The factors C/EBPα and PPARγ are activated during the differentiation process, and then PPARγ and C/EBPα together promote downstream adipose specific gene expression, which is involved in adipose phenotype, and also glucose and lipid metabolism [21,22].

In recent studies, anti-obesity effects have been shown for ginseng administration and the administration of several ginsenosides [23,24,25,26,27]. However, the physiological mechanisms underlying the anti-obesity effects of ginsenoside Rc are still not clear. In the present study, ginsenoside Rc was investigated for its effects on adipogenesis in mouse 3T3-L1 cells and the underlying mechanisms for this effect were investigated, including the PPARγ and C/EBPα pathways.

## 2. Results and Discussion

### 2.1. Ginsenoside Rc Inhibit 3T3-L1 Preadipocyte Proliferation

The effect of Rc on precursor adipose cells was determined using the MTT cytotoxicity assay. The results of the MTT assay indicated that 24, 48, 96, and 144 h exposure of cells to the Rc caused a concentration-dependent reduction of preadipocyte proliferation and viability (Table 1). The results shown in Table 1 revealed that the cellular oxidoreductase activity of 3T3-L1 cells treated with 6.25 μM of Rc for 48, 96, and 144 h was higher than those seen with the control at the end of 24 h, suggesting that Rc block the proliferation of 3T3-L1 cells. On the other hand, the cellular oxidoreductase activity of 3T3-L1 cells treated with 200 μM of Rc in 48, 96, and 144 h were lower than the values of the controls at the end of 24 h. And Rc were found to suppress the growth of the 3T3-L1 preadipocytes, with an IC50 of 427, 257, 186 and 157 μM for 24, 48, 96, and 144 h, which indicates that Rc may be toxic to 3T3-L1 cells at these high doses.

### 2.2. Ginsenoside Rc Was Effective in Inhibiting Adipocyte Differentiation

**Figure 1:**
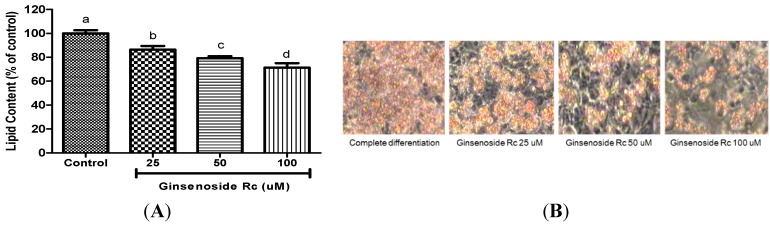
The effects of Rc on adipocyte differentiation (**A**) Post-confluent 3T3-L1 cells were differentiated in the absence or in the presence of Rc. Lipid content was quantified by measuring absorbance. Results are representative of three independent experiments, and statistically significant data are expressed as *p* < 0.05; (**B**) Lipid droplets were measured by Oil Red O staining. ^a–d^ Significant difference in treatment concentrating to ANOVA followed by Duncan (*p* < 0.05).

**Figure 2:**
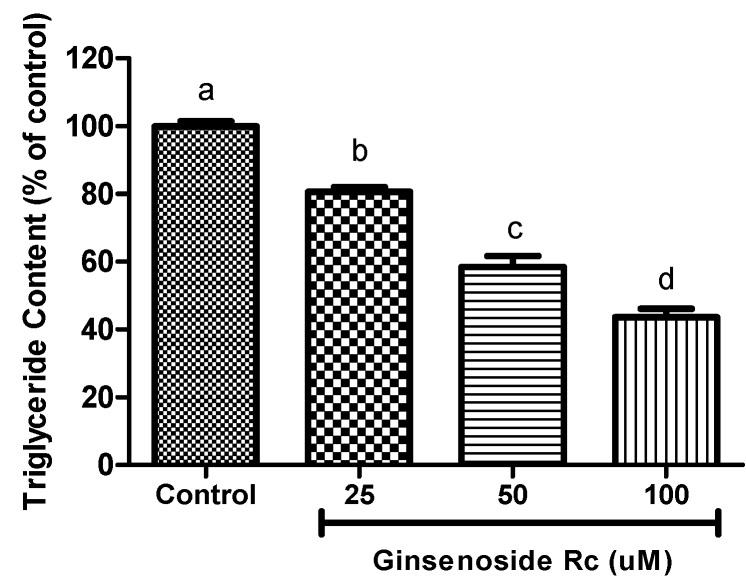
Effect of different Rc concentrations on the accumulation of triglyceide in adipocytes. Results are representative of three independent experiments, and statistically significant data are expressed as *p* < 0.05. ^a–d^ Significant difference in treatment concentrating to ANOVA followed by Duncan (*p* < 0.05).

Oil Red O staining was performed to examine the extent of lipid accumulation in 3T3-L1 adipocytes undergoing adipogenesis, either in the presence or absence of Rc. The representative images of Oil Red O staining shown in Figure 1 suggest that Rc suppressed lipid accumulation. It is evident from the data shown in Figure 2 that as the concentration of Rc is increased, and there is a proportional decrease in the rate of differentiation of 3T3-L1 preadipocytes—the differentiation rates at 25, 50 and 100 μM declined by 14%, 21% and 29%, respectively. It was noted that compared with the control group the level of differentiation decreases as the concentrations of Rc is increased. This result suggests that Rc treatment inhibits adipocyte differentiation.

**Table 1:** Effect of different concentrations of ginsenoside Rc on the proliferation of 3T3-L1 preadipocytes.

Treatment Concentration	Optical Density (λ = 595 nm)			
	24 h	48 h	96 h	144 h
Control	0.634 ± 0.017 ^aD^	0.808 ± 0.021 ^aC^	1.210 ± 0.025 ^aB^	1.503 ± 0.031 ^aA^
6.25 µM	0.599 ± 0.019 ^bD^	0.775 ± 0.019 ^bC^	1.125 ± 0.056 ^bB^	1.257 ± 0.065 ^bA^
12.5 µM	0.574 ± 0.021 ^bcD^	0.755 ± 0.018 ^bcC^	1.095 ± 0.043 ^bB^	1.211 ± 0.060 ^bcA^
25 µM	0.550 ± 0.011 ^cD^	0.725 ± 0.012 ^cdC^	1.055 ± 0.047 ^bcB^	1.157 ± 0.058 ^bcdA^
50 µM	0.553 ± 0.018 ^cD^	0.713 ± 0.012 ^deC^	1.007 ± 0.013 ^cdB^	1.124 ± 0.080 ^cdA^
100 µM	0.513 ± 0.010 ^dD^	0.698 ± 0.017 ^deC^	0.950 ± 0.053 ^dB^	1.072 ± 0.062 ^dA^
200 µM	0.460 ± 0.012 ^eC^	0.494 ± 0.020 ^eC^	0.564 ± 0.051 ^eB^	0.619 ± 0.019 ^eA^

^a–e^ Significant difference in treatment concentrating to ANOVA followed by Duncan (*p* < 0.05); ^A–D^ Significant difference in proliferating time to ANOVA followed by Duncan (*p* < 0.05).

### 2.3. Inhibition of Intracellular Triglyceride Content in 3T3-L1

Figure 2 shows the inhibitory effect of Rc on the accumulation of triglycerides in 3T3-L1 adipocytes. These results suggest that the inhibition of intracellular triglyceride accumulation in 3T3-L1 adipocytes occurred in a dose-dependent manner, when cells were exposed to Rc. Rc at concentrations of 25, 50 and 100 μM significantly inhibited triglyceride deposition in 3T3-L1 cells to 19.4%, 42.5% and 56.4%, respectively when compared to that of vehicle-treated cells at differentiation.

**Figure 3:**
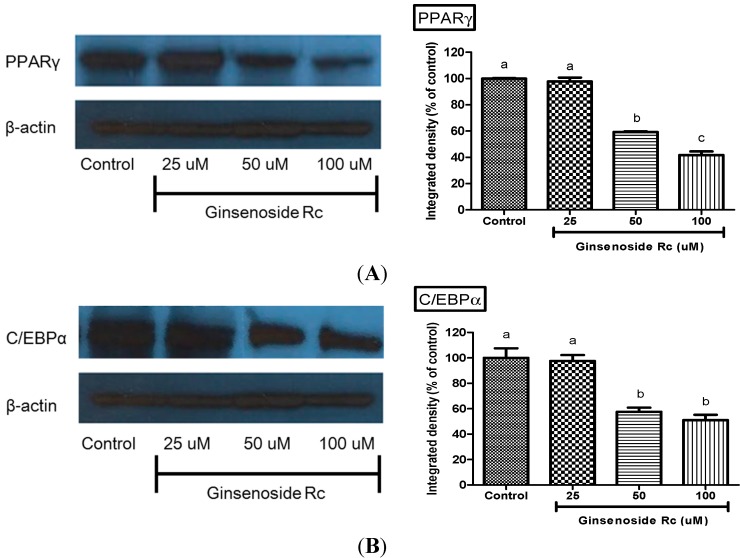
Effect of ginsenoside Rc on protein expression of (**A**) PPARγ; and (**B**) C/EBPα in 3T3-L1 cells. 3T3-L1 adipocytes were harvested 9 days after the initiation of differentiation. Expression was analysed by western blotting using specific antibodies. Data are presented as the mean ± standard errorof triplicate experiments. ^a–c^ significant difference in treatment concentrating to ANOVA followed by Duncan (*p* < 0.05).

### 2.4. Modulation of Adipocyte Differentiation-Related Protein by Rc

To investigate the inhibitory mechanism of Rc during the differentiation period, the expression of PPARγ and C/EBPα as transcription factors during adipocyte-differentiation was examined. The results shown in Figure 3 suggest that there is a significant dose-dependent decrease in the expression of leptin, PPARγ, and C/EBPa in 3T3-L1 preadipocytes, when induced to differentiate by Rc. The PPARγ protein expression levels in 25, 50 and 100 μM Rc-treated 3T3-L1 cells were 3%, 41% and 59% lower than those in control cells, respectively (Figure 3). The protein levels of C/EBPα in 3T3-L1 cells treated with 25, 50 and 100 μM Rc were decreased by 3%, 43% and 49% relative to expression levels in control, respectively (Figure 3). These results verify the potent inhibitory action of Rc on the differentiation of 3T3-L1 preadipocytes.

### 2.5. Discussion

Obesity is a contemporary global health concern associated with high morbidity and mortality. Accordingly, there is intense interest in developing an effective anti-obesity agent derived from natural products, whose safety have already been verified, rather than a synthetic substance whose safety would need to be verified. In recent years, many groups have explored the action of novel substances derived from natural sources in the context of adipogenic differentiation and gene regulation, at several stages associated with obesity [28], many studies have aimed to reduce obesity by focusing on decreasing preadipocyte differentiation and proliferation, and inhibiting lipogenesis, and increasing lipolysis. Ginseng has traditionally been used in Asian countries to improve health. The ginsenosides, of which there are various types, are the pharmacologically active components of ginseng [29]. Although the ginsenosides have been suggested to play roles in various diseases, such as diabetes, cardiovascular injury and cancer, their anti-obesity effects are poorly understood. In the last several decades, there is an emerging trend for the use of plant constituents to treat obesity and obesity-related diseases [30]. As part of our ongoing search to identify anti-obesity agents present in natural sources, we investigated the effects of ginsenosides on adipocyte differentiation and lipid oil formation in 3T3-L1 pre-adipocytes [31,32,33].

The 3T3-L1 cell line is commonly used as an adipocyte differentiation model system for investigating the molecular mechanisms of adipogenesis. To examine the effects of the ginsenoside Rc on the differentiation of preadipocytes into adipocytes, confluent 3T3-L1 preadipocytes were treated with various concentrations of samples during differentiation. The differentiated adipocytes were stained with Oil-Red O solution, and their lipid contents were quantified spectrophotometrically. The Rc were found to significantly reduce lipid accumulation in 3T3-L1 preadipocytes compared with a vehicle control group. Our data revealed that the Rc possess promising anti-obesity effects, including inhibiting adipocyte differentiation and lipid formation in 3T3-L1 cells. Capsaicin and 6-gingerol has previously been reported to possess anti-adipogenic activity and cytotoxicity on 3T3-L1 cells [34,35]. Rb1 inhibited cellular triglyceride accumulation in 3T3-L1 adipocytes [31].

Subsequently, we investigated the effects of Rc on the adipocyte differentiation of 3T3-L1 cells by determining the expression levels of key adipocyte marker genes including PPARγ and C/EBPα. These markers C/EBPα and PPARγ are two adipogenic transcription factors that play key roles in adipocyte differentiation [20,21,22]. Expression of these two factors is elevated during differentiation process and required for the survival of the mature adipocyte. Overexpression of PPARγ and C/EBPα can induce and accelerate adipocyte differentiation [20]. Generally, adipocyte differentiation involves a series of transcriptional processes. The first stage of adipogenesis consists of the transient induction of C/EBPβ and C/EBPδ and can be stimulated *in vitro* by hormonal differentiation [36]. Next, C/EBPβ and C/EBPδ begin to accumulate and both directly induce expression of PPARγ and C/EBPα, the key transcriptional regulators of adipocyte differentiation [37]. PPARγ and C/EBPα initiate positive feedback to induce their own expression and also to activate a large number of downstream target genes whose expression determines the adipocyte phenotype. In addition, C/EBPα induces many adipocyte genes directly, and *in vivo* studies have indicated an important role for this factor in the development of adipose tissue. On the other hand, PPARγ is a member of the nuclear receptor super-family of ligand-activated transcription factors and is a prerequisite for the differentiation of adipocytes [38]. All studies performed in PPARγ function models have confirmed that PPARγ is both necessary and sufficient for fat formation [39]. The expression of C/EBPα in fibroblasts can induce adipogenesis only in the presence of PPARγ [40]. Accordingly, PPARγ expression can induce adipogenesis in mouse embryonic fibroblasts of C/EBPα deficiency, but C/EBP-α cannot rescue adipogenesis when PPARγ is not expressed, suggesting that PPARγ is a master regulator of adipogenesis [41]. No factor has been discovered to promote adipogenesis in the absence of PPAR-γ, and most pro-adipogenic factors seem to function at least in part by activating PPARγ expression or activity. Thus, PPARγ is required for maintenance of the differentiated state. Commonly, PPARγ is expressed at an early stage of cell growth (days 1–2 post-confluence), whereas C/EBPα is expressed at the intermediate stage (day 4 post-confluence). These transcription factors further control the expression of adipocyte-specific genes in the late stage (day 6 post-confluence), thereby leading to fat droplet formation [42]. Our results showed that the Rc significantly attenuated the expression of PPARγ and C/EBPα compared with adipocytes, indicating that the Rc inhibited adipogenesis in the 3T3-L1 cells by down-regulating PPARγ and C/EBPα expression. Rc-mediated inhibition of PPARγ and C/EBPα at the early/intermediate stages results in the suppression of adipocyte-specific genes and lipid formation observed at the later stage of adipocyte differentiation. The lipid accumulation was negatively correlative to the protein levels of PPARγ and C/EBPα in these cells treated with different concentrations of Rc. Clearly, these results imply that Rc has the ability to suppress adipocyte differentiation through the signaling pathways involving PPARγ and C/EBPα. Rf and Re have been reported to have anti-adipogenic activity through the down-regulation of PPARγ [32,33]. Rb1 promoted adipogensis by enhancing C/EBPα and PPARγ expression in a concentration-dependent manner [31]. These results suggested that Rc is promotes anti-adipogenic activity on 3T3-L1 adipocytes by down-regulating C/EBPα and PPARγ.

## 3. Experimental Section

### 3.1. 3T3-L1 Cell Culture and Adipocyte Differentiation

The mouse embryo 3T3-L1 cell line was obtained from the American Type Culture Collection (Manassas, VA, USA). The 3T3-L1 preadipocytes were grown in Dulbecco’s Modified Eagle’s Medium (DMEM; GIBCO BRL Life Technologies, Invitrogen Corporation, Carlsbad, CA, USA), supplemented with 10% heat-inactivated (56 °C, 30 min) newborn calf serum (NCS; 26010-074, GIBCO BRL Life Technologies, Invitrogen Corporation) and antibiotics (100 units/mL penicillin and 100 g/mL streptomycin). The culture was maintained at 37 °C in a humidified atmosphere, containing 5% CO_2_ and 95% air. For adipocyte differentiation, the 3T3-L1 cells were grown in 6-well plates inoculated at 4.5 × 105 cell/cm^2^ to confluence. Two days post-confluent, preadipocytes were stimulated by a differentiation mixture containing 1 μM dexamethasone (DEX; Sigma-Aldrich, Louis, MO USA), 0.5 mM 3-isobutyl-1-methylxanthanine (IBMX; Sigma-Aldrich) and 1 μM insulin in DMEM with 10% fetal bovine serum (FBS). After 2 days the medium was replaced with DMEM containing 10% FBS and 10 μg/mL insulin. Cultures were incubated for 2 further days. Subsequently the culture medium was again replaced with DMEM/10% FBS and refreshed at 2 day intervals thereafter until analysis was performed on days 6–8. By about day 10 to day 14, about 90% of the 3T3-L1 cells differentiated in adipocytes showing typical visual.

### 3.2. MTT Assay

The MTT assay was performed according to the method of Mosmann [43]. This assay is based the mitochondrial dependent reduction of 3-(4,5-dimethylthiazol-2-yl)-2,5-diphenyl tetrazolium bromide (MTT, Sigma-Aldrich) to formazan, which is directly proportional to the number of living cells in culture. 3T3-L1 preadipocytes were plated into 96 well plates a density of 1 × 104 cells/well. After 24 h, the culture medium was replaced by 100 μL serial dilutions (0.01–1000 μg/mL) of sample and the cells were incubated for 24, 48, 96 and 144 h. Culture supernatants were then removed and replaced by 90 μL of fresh culture medium. 10 μL of a sterile, filtered MTT solution (5 mg/mL) in phosphate-buffered saline (PBS, pH = 7.4, Invitrogen, Carlsbad, CA, USA) was added to each well to reach a final concentration of 0.5 mg MTT/mL. After 5 h, the unreacted reagent was removed, and then insoluble formazan crystals were dissolved in dimethyl sulfoxide (100 μL/well) and absorbance at 595 nm was measured using a microplate reader (Bio-Rad, Philadelphia, PA, USA).

### 3.3. Determination of Lipid Accumulation by Oil Red O Staining

Ginsenoside Rc at a concentration of 25, 50 and 100 mM, was added to the medium at each of the three stages of adipocyte differentiation. The lipid content in the mature adipocytes was determined using the Oil Red O staining method. The cells were fixed with 10% formalin for 1 h, washed with 60% isopropanol, and completely dried. Subsequently, the cells were stained with Oil Red O solution in isopropanol for 10 min, and then washed four times with water. The images of the Oil Red O stained cells were acquired using an inverted contrast phase microscope (Axiovert 40 CFL, Carl Zeiss, Jena, Germany). Fat droplets stained red were extracted from cells using isopropanol and the absorbance was measured at a wavelength of 540 nm.

### 3.4. Measurement of Triglyceride Content

3T3-L1 adipocytes that were harvested 12 days after the initiation of differentiation were incubated with ginsenoside during the differentiation process in six-well plates at 37 °C in a humidified 5% CO_2_ incubator. Cells were collected and lysed using an Ultrasonic Cell Disruption System (IKA^®^-Werke GmbH & Co. KG, Staufen, Germany). The total TG content in the cells was determined using the TG assay Kit. (Cayman Chemical Company, Ann Arbor, MI, USA).

### 3.5. Western Blot Analysis

3T3-L1 preadipocytes were seeded on 6-well plates and left to differentiate for 10–14 days prior to treatment with various doses of ginsenosides Rc for 2 or 24 h. The cells were pretreated for 1 h with cycloheximide (5 μg/mL) before ginsenosides were added, to block protein synthesis. The cells were then washed with ice-cold PBS and lysed in 0.2 mL of lysis buffer (1% NP-40, 150 mM NaCl, 0.1% SDS, 50 mM Tris-HCl, pH 7.6, 10 mM EDTA, 0.5% deoxycholate, 1 mM PMSF, 1 mM sodium orthovanadate, 10 mM NaF, 10 mM β-glycerophosphate, 10 μg/mL protease inhibitor, and phosphotase inhibitor cocktails) for 30 min at 4 °C. Equal amounts of protein of each sample were separated by sodium dodecyl sulfate (SDS)-10% polyacrylamide gel electrophoresis (PAGE) and transblotted onto polyvinylidene difluoride (PVDF) membranes (Millipore, Bedford, MA, USA). Immunoblotting was performed with antibodies for PPARγ, C/EBPα and β-actin antibodies were from Cell Signaling Technology (Beverly, MA, USA). Signals were visualized with an enhanced chemiluminescence kit (Amersham Biosciences, Buckinghamshire, UK) according to the manufacturer’s instructions, followed by exposure to X-ray films.

### 3.6. Statistical Analysis

All experiments were performed in triplicate. The results obtained were expressed as mean ± SD. Statistical analysis was performed using SAS software. Analysis of variance was performed. Significant differences (*p* < 0.05) between the means were determined by Duncan’s multiple range tests.

## 4. Conclusions

In conclusion, our results demonstrated that ginsenoside Rc efficiently inhibited adipogenesis in 3T3-L1 adipocytes in a dose-dependent manner as indicated by a significant reduction in intracellular triglyceride contents and lipid accumulation, without eliciting apparent cytotoxicity. Furthermore, the suppressive effects of Rc were possibly mediated by down-regulated expression of major adipogenic transcription activator (PPAR-γ and C/EBP-α) proteins of the adipogenesis pathway. Collectively, these findings provide strong evidence that Rc might inhibit adipogenesis through downregulation of PPARγ and C/EBPα expression in 3T3-L1 adipocytes. Therefore, Rc may provide a possible therapeutic approach for the prevention and treatment of obesity. Further *in vivo* research and clinical trials are needed to clarify the efficacy, safety, and precise molecular mechanisms of the anti-obesity effects of these ginsenosides.
